# Perfluoro-n-octane mimicking an intraocular foreign body

**DOI:** 10.3205/oc000081

**Published:** 2017-12-15

**Authors:** Prabu Baskaran, Pratyusha Ganne, Nagesha C Krishnappa

**Affiliations:** 1 Department of Vitreo-Retina, Aravind Eye Hospital and Postgraduate Institute of Ophthalmology, Pondicherry, India

**Keywords:** perfluoro-n-octane, intraocular foreign body, rodio-dense, CT scan

## Abstract

Retained intraocular foreign body (IOFB) is a major cause of visual loss following open globe injuries. Detecting the presence and accurate localization of IOFB in the setting of an open globe injury remains a challenge. There can be various mimics of intraocular IOFB on imaging including air, ocular calcifications, etc. Here, we describe a case of open globe injury wherein a retained perfluoro-n-octane bubble mimicked a retained intraocular foreign body.

## Introduction

Retained intraocular foreign body (IOFB) is a major cause of visual loss following open globe injuries. Studies have shown that up to 40 percent of open globe injuries can have retained IOFB [1]. Loss of vision can result from endophthalmitis, retinal detachment or metallosis. Hence, IOFB mandates emergency removal. Detecting the presence and accurate localization of IOFB in the setting of an open globe injury remains a challenge. The various modalities used include computed tomography scan (CT), magnetic resonance imaging, ultrasonography, and plain film x-ray. The sensitivity and specificity for detection depends on the imaging modality and the type of IOFB [2]. There can be various mimics of intraocular IOFB on imaging including air, ocular calcifications, etc. 

## Purpose

To report a case of open globe injury wherein a retained perfluoro-n-octane bubble mimicked an intraocular foreign body necessitating a second surgery. 

## Case description

**Figure 1:**
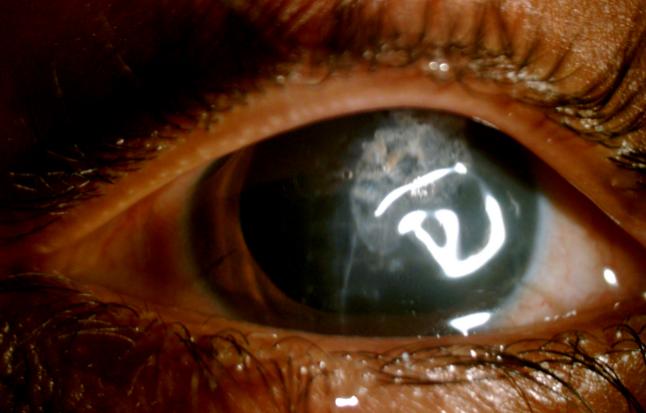
Clinical photograph of the left eye following corneal tear repair with fibrin glue. Note the central large wound.

**Figure 2:**
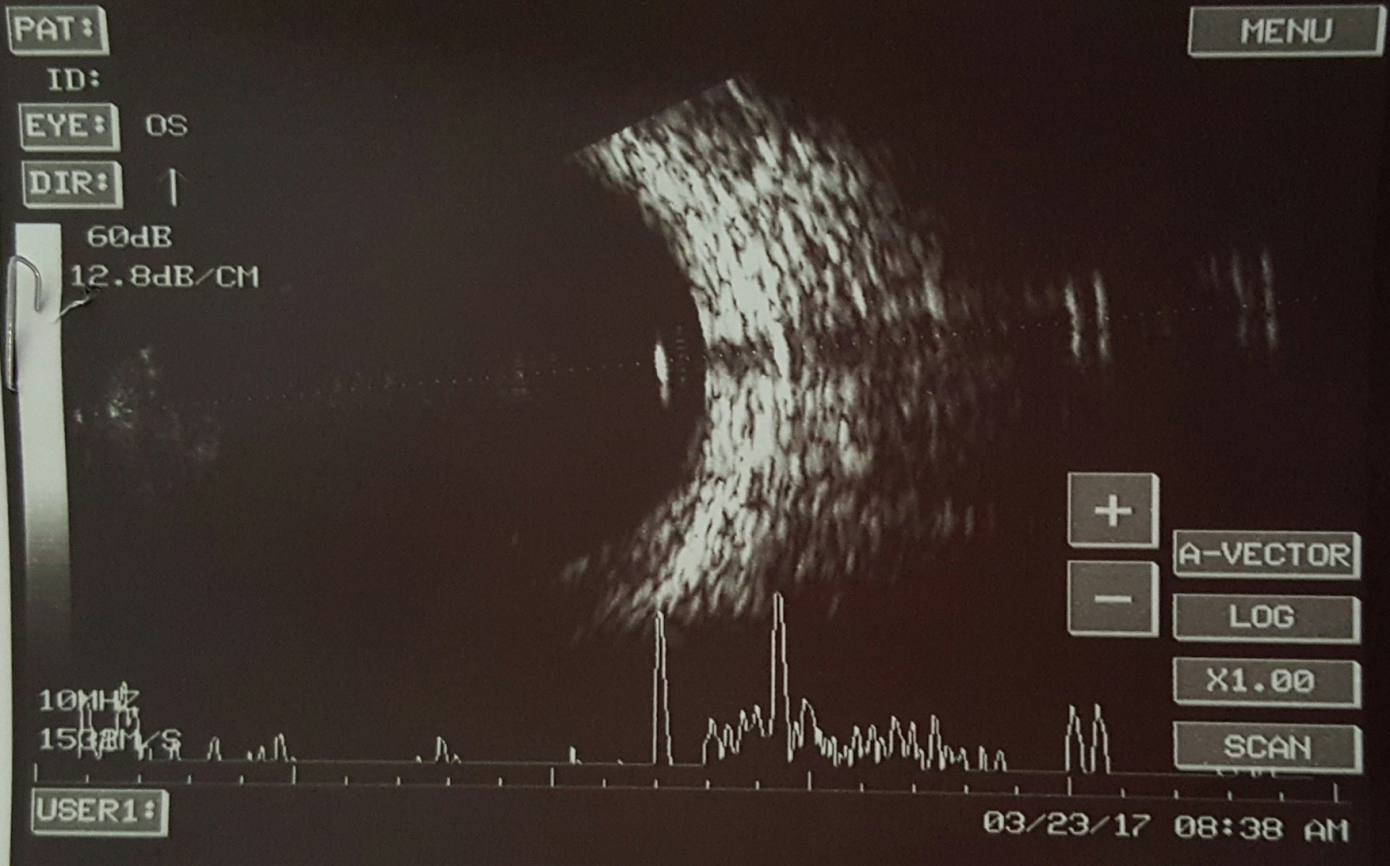
Ultrasonography following the initial surgery showing a hyperintense lesion with backshadowing suggestive of a foreign body

**Figure 3:**
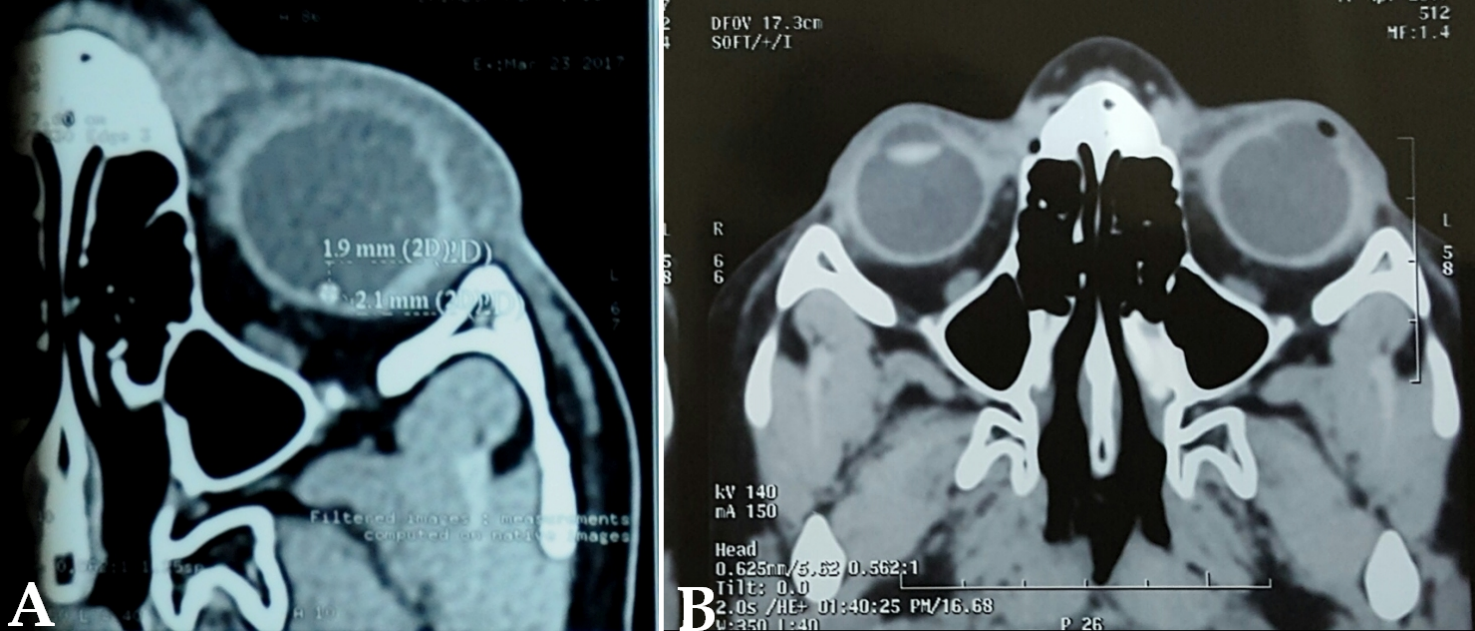
A: CT scan image after the initial surgery showing a round, 1.9 mm x 2.1 mm sized radio dense (400 Hounsfield Units) lesion close to the retina in the left globe. B: CT scan following aspiration of the PFCL bubble showing the absence of any radio dense lesions.

A 10-year-old boy presented to the emergency with a history of blast injury. His visual acuity at presentation was 6/6 in the right eye and hand movements in the left. Ocular examination showed a full thickness corneal laceration measuring 10 mm passing through the center of the left cornea. He underwent corneal tear suturing with lens extraction as an emergency procedure by the cornea team (Figure 1 (Fig. 1)). Intraoperatively, there was a posterior capsular rent with cortex drop which necessitated a pars plana vitrectomy. During pars plana vitrectomy, three non-metallic foreign bodies ranging in size from 0.5 to 2 mm were removed from the posterior segment. Perfluoro-n-octane was used to assist the surgical removal. The visibility throughout the surgery was poor due to the corneal wound. On the first postoperative day, the posterior segment could not be examined clinically due to corneal edema and astigmatism. Ultrasonography of the posterior segment revealed a high reflective echo with back shadowing in the posterior vitreous (Figure 2 (Fig. 2)). A plain computed tomography scan (CT) was done which revealed a round, 1.9 mm x 2.1 mm sized radio dense (400 Hounsfield units) lesion close to the retina in the left globe (Figure 3A (Fig. 3)). With the suspicion of a retained intraocular foreign body, the patient was taken up for a repeated pars plana vitrectomy. Intraoperatively, there was no evidence of a foreign body. Instead, there was a small bubble of perfluoro-n-octane over the posterior pole. The bubble was aspirated. CT scan was repeated in the postoperative period which showed the absence of any radio dense lesion (Figure 3B (Fig. 3)).

## Discussion

Penetrating injuries with a retained intraocular foreign body are an important cause of blindness among children. Studies have shown that the removal of intraocular foreign bodies within 24 hours from the time of trauma significantly reduces the risk of endophthalmitis [3]. CT scan is the modality of imaging for the detection of IOFB. Different foreign bodies produce different degrees of attenuation on a CT scan. Metallic foreign bodies cause the highest degree of attenuation while wood causes the lowest [2]. Perfluoro-n-octane has been used to aid complicated vitreoretinal surgeries. It is a high density, synthetic fluorinated compound. Although it is completely removed at the end of surgery, small amounts may be retained especially when the visibility during the surgery is poor. Owing to their high density, they show up as hyperintense lesions on ultrasonography and radio dense lesions on a CT scan [4]. In the present case, the visibility during the first vitrectomy was poor and multiple foreign bodies were removed during this time. Hence, there was a high index of suspicion of a missed foreign body. This subjected the child to an unnecessary second surgery with attendant risks of general anesthesia. Liu et al. [5] have published a similar case report of retained perfluorodecalin masquerading as a retained intraocular foreign body. The authors differentiated the bubble from an IOFB by its ability to move freely, its smooth contour and the lack of any metallic streak artifact. 

This case emphasizes the need to exercise caution when making a diagnosis of an IOFB especially when the contours of the foreign body are smooth and regular.

## Notes

### Competing interests

The authors declare that they have no competing interests.
